# Examination of the proposed relationships between cyclic menstruation and human reproduction

**DOI:** 10.3389/fgwh.2025.1589736

**Published:** 2025-07-07

**Authors:** Vernon G. Thomas

**Affiliations:** Department of Integrative Biology, College of Biological Science, University of Guelph, Guelph, ON, Canada

**Keywords:** food security, ovary, progesterone withdrawal, hormonal inhibition, endometrium, parturition, old world primates

## Abstract

Increased societal food security has enabled frequent ovulation based on women's body fat levels remaining above the fertility threshold. The consequential cyclical menstruation in non-pregnant women constitutes a large female health and welfare issue. This examination of the various roles proposed for menstruation could not detect an essential biological role, whether in the removal of un-implanted embryos or the conditioning of the endometrium against oxidative stress. Neither is menstruation a prerequisite for the regeneration of the uterine endometrium. Menstruation and foetal placental detachment from the endometrium at birth share common hormonally-controlled physiological processes initiated by systemic or local progesterone withdrawal. These critical genetically-conserved processes are essential for the survival of both infant and mother. In human ancestors menstruation likely occurred rarely and these processes were activated only during the birth of several children. Now, because of assured food security, the evolved ancestral function of these processes has been reversed, and even allowing for the birth of several children, a woman may experience hundreds of monthly periods in her life. Menstruation is not necessary for a healthy life and women who control hormonally their periods and fertility still retain the physiological capacity for foetal placental detachment, to be expressed should pregnancy occur. This examination's findings could reduce the ignorance and mis-understanding about menstruation and promote, globally, social policy to reduce “period poverty” and absenteeism of girls and women from schools and the workplace.

## Introduction

Despite advances in the education of society about menstruation, ignorance, prejudice, and myths persist (1) that deny the relevance and importance of menstruation and its related health impacts on the lives of girls and women. Half of the World's humans now menstruate for about a quarter of their fertile life of approximately 35 years, so the biological significance of this event might have emerged long ago, especially given the vast number of physiological-biochemical studies related to it. Women may now experience over 400 menstrual periods during their life (2). A third of women report varying degrees of physical discomfort during their monthly periods. A quarter of women suffer severe debilitating conditions related to heavy uterine bleeding, and a further subset experience abnormal uterine bleeding (3). This is the most serious chronic condition affecting women's health and welfare, globally, and especially women in low and middle-income countries (4, 5).

Addressing and alleviating these debilitating conditions is confounded by diverse views regarding the relationship between menstruation, reproduction, and health, especially among the scientific-medical and non-scientific communities. This perspective addresses and investigates those conflicting views. In 2022, the World Health Organization (6) called for menstrual health to be recognized and addressed as a health and human right issue, not a hygiene issue, a view endorsed by Martin et al. (5). However, Critchley et al. (7) stated that realizing these goals requires a better interpretation of how menstruation relates to both health and reproduction.

The uterus is the most anatomically-dynamic organ in the female body, the cellular matrix of the endometrium having a life span of only ∼4 weeks (8) in response to the continuously fluctuating oestrogen and progesterone releases from the ovarian follicle and especially the withdrawal of progesterone that initiates menstruation (3, 9). Is it possible that the various debilitating conditions of heavy uterine bleeding and its associated anemia that often accompany menstruation (10) are related to the cyclical rapidity of the shedding and repair of the uterine lining, a phenomenon to which it is ancestrally unaccustomed? Women now experience far more periods than their ancestors, experiencing more than 400 menses compared to as few as 40 menses over a reproductive life course (2, 11). Perhaps we have asked the wrong questions about menstruation by focussing only in the context of the non-pregnant woman. Thus, a wider examination of human reproduction is required in which the origin of cyclical menstruation is examined and whether it serves an essential reproductive function. Comparisons with women living presently as hunter/gatherers under early Stone Age conditions are not possible, but several species of wild Old World primates (the Great Apes) that have the capacity for menstruation serve as valid comparisons. It is also important to consider two critical events in the 9-month life of the human embryo. One is successful implantation of the embryo in the uterus. The other is how the foetal placenta detaches from the uterus without impairing the life of the mother. The roles of progesterone, both in developing the uterine endometrium prior to implantation and its withdrawal in initiating menstruation and parturition (3, 12, 13), are central to this article. A brief account of how cyclical menstruation arose is required prior to examination of its proposed roles.

## Food security, nutrition, and reproduction

Cyclical (or continual) menstruation likely arose with women attaining food security, a prominent feature of human cultural evolution. Hunting, fishing, and gathering progressed to pasturalism, coupled with animal husbandry. Later, the Agricultural Revolution, augmented by the Industrial Revolution, increased both the scale and level of global food production and availability. In the past Century, fossil fuels enabled the synthesis of organic herbicides, pesticides, and nitrogen-based fertilizers leading to the Green Revolution and industrialisation of modern agriculture. Now foods are transported internationally, aided by modern systems of food preservation.

Nutrition has profound effects upon human health and reproduction (11). Early Hominids were, likely, seasonal breeders whose females spent most of their adult lives between pregnancy and lactation, with few, if any menstrual periods (2, 11). Later, reliable food supplies allowed cyclical reproduction to develop as more females maintained their body fat level above the approximately 27% fertility threshold (14, 15). Nutrition's importance to reproduction was emphasized by Ellison who indicated that fertility (ovarian function) is subject to metabolic regulation in which steroid production depends on energy availability (16). Possibly, related to this, the age of menarche has moved to the pre-teenage years (17), and access to high-quality food has decreased the interval between successive births. However, should the body fat level of adult women fall indefinitely below the fertility threshold due to inadequate nutrition and/or prolonged intense physical activity, both ovulation and menstruation cease. Apart from nutritional considerations, a non-pregnant life without menstruation is becoming more popular among women choosing to avoid the inconvenience and discomfort of monthly periods by control of their menstrual experience with hormonal preparations (18).

## The nature of menstruation

**Figure 1:**
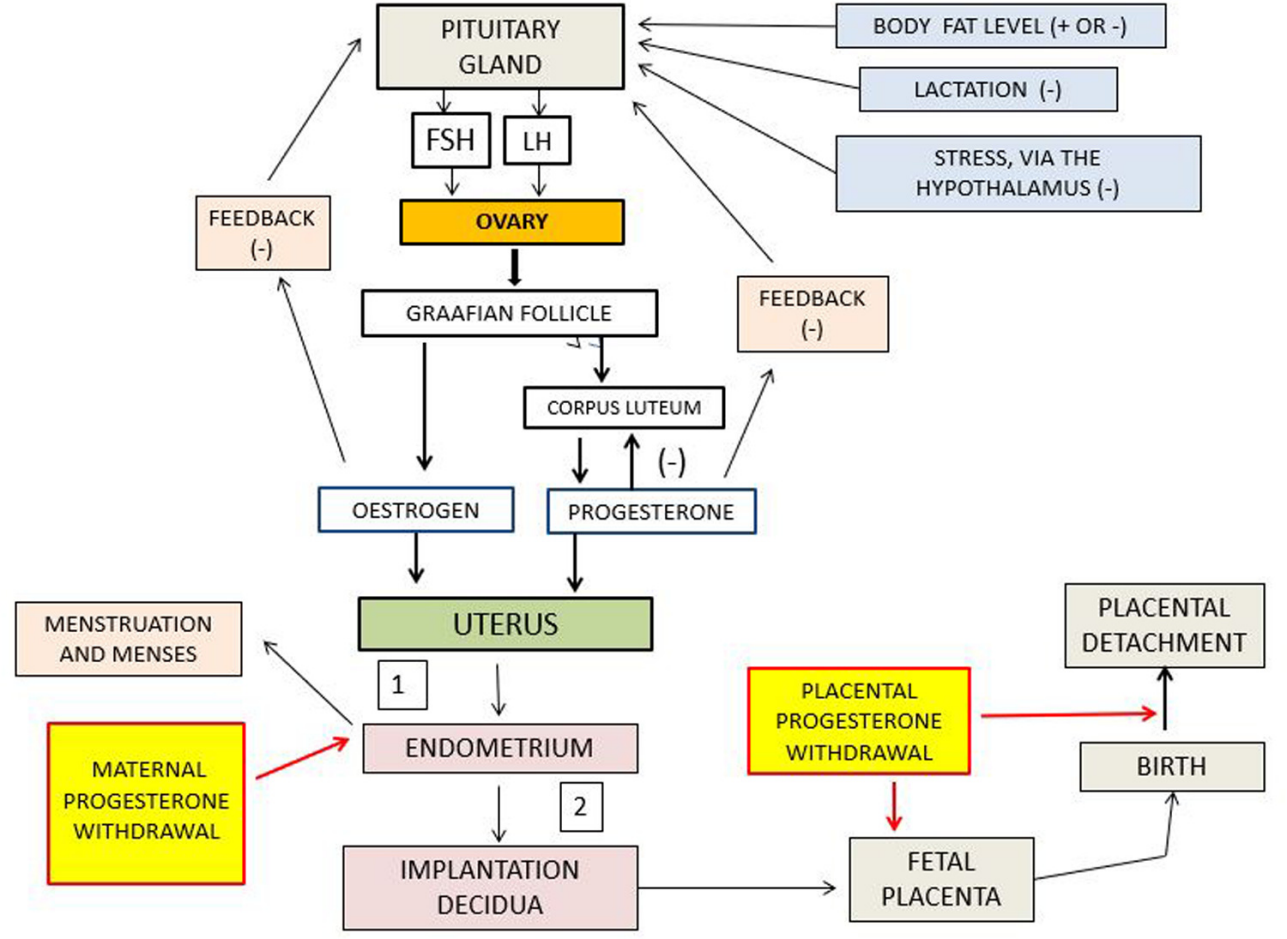
Schematic representation of the interactions among the pituitary, ovary, and uterus. Positive and negative signs indicate stimulation and inhibition, respectively. 1 denotes failed conception leading to menstruation, and 2 denotes pregnancy leading to parturition. The red lines indicate the paths of impact of withdrawal, or lowering, of progesterone. FSH is follicle stimulating hormone: LH is luteinizing hormone. Note that in this scheme there is no feedback between menstruation and the endometrium.

Menstruation evolved independently in several groups of placental mammals (Eutheria) (19), but only recently in Old World primates of Africa and Eurasia. It reaches its greatest extent in human beings in whom a haemochorionic placenta develops in which the implanted embryo invades the uterine endometrium (termed the decidua post-implantation) to produce an intimate relation with the maternal blood by eroding the cell walls of the uterine arterioles. The extent of decidua invasion by the implanted embryo is largest in humans (20). This placental arrangement results in a vast surface area over which maternal-foetal exchange can proceed. The decidua fulfils a dual role, enabling the fetus to acquire enough oxygen and metabolites until the end of pregnancy while, simultaneously, enabling the mother to survive and have further pregnancies. Should conception or embryonic implantation not occur, the endometrium/decidua is shed from the uterine surface as menses. Menstruation is initiated by a falling level of circulating progesterone (Figure 1) in response to the regression of the ovarian corpus luteum, and begins with a constriction of the spiral arterioles of the endometrium (3). Cyclical menstruation occurs when the repeated regeneration of the uterine endometrium follows the onset of menstruation. The reproductive health of women who control their menstrual bleeding experience via hormonal contraceptives is not jeopardized (18), and menstrual bleeding suppression is completely reversible when use of hormonal contraceptives stops.

## Examination of proposed roles for menstruation in human reproduction

Diverse explanations of menstruation have been proposed, based on the assumption that it has a biological purpose that requires identification. Finn (21, 22) stated that menstruation is a non-adaptive inevitable consequence of failed conception which, isolated from reproduction, has no evolutionary future. However, Travis (23) suggested that it must be purposeful, given its appearance in all present-day women. The proposed explanations for an essential role of menstruation now require examination.

The interpretations of Profet (24), that menses serve to rid the genital tract of pathogens derived from males during copulation, and Strassmann (25), that shedding of the uterine lining is more energy efficient than its costs of maintenance, have been criticized severely and discounted by Finn (22, 26), Clarke (27), Clough (28), Martin (29), and Emera et al. (30).

A role of the uterus in detecting embryos of “low quality” upon implantation as the basis of menstrual evolution was advanced by Haig (31). Clarke (27) hypothesized that the uterus selects embryos that are unable to develop and that menstruation is the means to remove them from the uterus. This concept of the uterus playing an active role was developed further by Teklenburg et al. (32), Macklon and Brosens (33), and Brosens et al. (34) who suggested that the endometrium senses embryo quality and that the decidualisation (i.e., the maximal development of the endometrial tissues) preceding menstruation is the mechanism to identify, and then remove, embryos incapable of normal development. However, the physiological mechanism by which “normal” and “abnormal” embryos are selected has not been identified. Teklenburg et al. (32) observed that impaired embryos trigger a reaction from the endometrium, and that shedding of the uterine endometrium evolved to offset the high rate of chromosomal abnormalities in human embryos.

Wasser and Barash (35) reported that 50%–60% of first trimester human embryos display errors in chromosomal separation (or disjunction) occurring at conception, producing 10 recognized trisomous conditions (an extra chromosome from either parent), some of which lead to early embryonic death. There are also many (over 6,000 [GARD (36)] cases of inherited human genetic diseases where the concept of a selective uterus fails and in which, what may be considered by some, as deleterious genes are passed to children and adults. Expecting the uterus to be an arbiter of natural selection runs counter to all evolutionary theory because evolution is neither anticipatory, purposeful, nor precautionary. The criteria upon which an uterus selects have not been specified, except that both negative and positive signals at the embryo-uterine surface combine to detect normal and abnormal embryos. In those cases where the embryo fails to develop, a distinction must be made between the elimination of an abnormal embryo and the deliberate rejection of that embryo by the uterus.

An extensive chemical communication between the embryo and the uterine decidua is required for successful implantation. This involves 21 distinct genetic interactions (37) vital among which is the early production of the embryonic hormone chorionic gonadotropin (HCG), so critical for successful implantation (38). An abnormal embryo that fails to produce HCG does not implant and does not control the ovarian follicle's release of progesterone (39), in the absence of which menstruation occurs. At the following stage of embryonic development (weeks 7–9 of gestation), failure to effect the transition from chorionic gonadotropin control of the corpus luteum to placental production of oestrogen and progesterone (the luteo-placental shift) (40, 41) also results in an early pregnancy loss and eventual menstruation. The un-implanted embryo is then removed from the uterus presumably by processes that also remove superfluous sperm in the oviduct post-fertilization (42). Components of the immune system are always present at the eyes, nose, and respiratory tract, among other locations in the body, to protect against invasive pathogens. Shedding of the entire uterine surface as menses over several days is not required to remove a single 8–16 cell un-implanted embryo. Menstruation is the consequence of failure of the embryo to implant, not uterine selection.

Another explanation for cyclic human menstruation suggests that it preconditions the uterus by protecting it from the subsequent oxidative stress related to deep embryonic invasion (43). It is hypothesized that repeated regeneration of the endometrium tissues after menstruation protects a subsequent decidua from excessive inflammation and stress that can accompany deep embryonic implantation. In this sense, cyclical menstruation would be beneficial and could, hypothetically, moderate various placental disorders attributable to excessively deep implantation (44).

### Comparison with non-menstruating mammals and old world primates

Most of the explanations for menstruation proposed recently deal with developmentally-impaired human embryos. Chromosomal and genetic aberrations in embryos are not unique to human beings, and must occur in other mammals that do not menstruate. Then, the covert elimination of the abnormal embryo is assumed to occur, as it is proposed now for humans. The monthly rapid regeneration of the endometrium, as opposed to only following birth, was viewed as an adjunct to successful human reproduction (45, 46). However, females in wild Old World primate species normally conceive during their first oestrus, suggesting that menstrual “rehearsals” are not a pre-requisite for successful reproduction. This argues against the roles of cyclical menstruation in uterine conditioning and removal of un-implanted embryos proposed by Brosens et al. (43) and Blanks and Brosens (45). While repeated menstrual cycles may help alleviate signs of the “great obstetrical syndromes” in humans (44), such syndromes would not persist in wild primate females who failed to leave offspring. Moreover, cyclical menstruation arises from successive failures of conception (i.e., failed reproduction), so this cannot be the evolutionary selection factor for human menstruation. A wild primate female who experienced several menstrual periods would have consumed food that otherwise would have been used for producing offspring. That female would also have benefitted from being part of a group. Natural selection would not favour a mature female who passed several years without breeding and failed to pass her genes to successive generations, quite apart from not adding numerically to the size of her group. Emera et al. (30) proposed that “conflict” arising between the blastocyst and the endometrial surface was the cause of menstruation evolving in Old World primates. Genetic discord, meaning differences in the genetic contributions of the parents to an embryo, is assumed to be a normal consequence of non-incestuous human reproduction, and all sexual reproduction. All normal mammalian embryos, having the capacity to develop to term, must still avoid rejection by the female because of their allogenic property. This aspect of reproduction affects all species of placental mammals, especially those with haemochorionic placentas, but is not a peculiar feature of human reproduction requiring menstruation to resolve.

### Examining the view that menstruation is essential for successful conception

Many women conceive after many menstrual periods have occurred, giving rise to the view that menstruation is a precursor for pregnancy; the removal of the decidua being necessary for endometrial regeneration (22). In women who menstruate continually it is self-evident that endometrial regeneration naturally follows menstruation. Here, it is critical to distinguish between menstruation occurring as a *consequence* of progesterone withdrawal and menstruation existing, *purposefully*, to initiate regeneration of the endometrium. It is also important to distinguish between the “capacity” for menstruation, as in the developed endometrium, and the “expression” of menstruation as in uterine shedding. At the same time that progesterone withdrawal initiates endometrial shedding, the same progesterone withdrawal is removing inhibition of the pituitary-ovarian axis, so leading to the development of a Graafian follicle (Figure 1). This is the essential component of the human reproductive cycle. The view that menstruation is critical for subsequent development of the endometrium overlooks the fact that endometrial regeneration depends entirely on endocrine stimulation from a functioning Graafian follicle. That stimulation cannot be provided from the degenerated products of menstruation. The newly-stimulated Graafian follicle releases oestrogen and (later) progesterone that, collectively, enable regeneration of the endometrium. It is the nature of the corpus luteum-progesterone feedback loop that (in conjunction with oestrogen) determines the development of the endometrium and its eventual demise (Figure 1). Removal of pituitary inhibition of the ovary should be considered the critical event because it leads to the next phase in the monthly cycle (i.e., endometrial development), whereas menstruation leads only to endometrial shedding.

Menstruation is then an *inevitable consequence* of progesterone withdrawal. Possibly, because menstruation is so blatantly visible and readily investigated compared to progesterone lowering, it has been accredited with a function that ought to have been attributed to progesterone withdrawal. It is difficult to understand how shedding and permanent loss of the endometrial tissue promotes the simultaneous regeneration of the same tissue at the same location. Also, constriction of the spiral arterioles adjacent to the menstruating endometrium prevents a hypothetical feedback to the ovary via the uterine veins.

It is also important to address the view (however, not supported by the scientific literature) that while life without menstruation is possible, such women cannot reproduce. That view is correct *at the time of amenorrhea* when the ovary is inhibited from developing follicles and releasing hormones. However, this situation is reversible (depending on the cause of the amenorrhea) and fertility then resumes. When menstruation does not occur because of inadequate maternal nutrition, or intense physical-metabolic demands, inhibition of the pituitary-ovarian axis occurs (Figure 1). Resumption of adequate nutrition restores ovarian function, including the development of the uterine endometrium. Pregnancy is then possible, but there has not been a preceding menstrual event to make it possible. Ovarian function is also inhibited during lactation (Figure 1) following birth. When lactation ceases, normal ovarian functions are resumed leading to the development of the endometrium. Again, in this situation, there has been no preceding menstruation.

In wild Old World primate populations whose reproduction is seasonal, reproduction is initiated by endogenous and exogenous factors that, together, activate the ovary, leading to endometrial development. As indicated previously, there would be evolutionary selection against females that required menstruation to develop a functional decidua. However, where a woman has suppressed menstruation by hormonal preparations the pituitary-ovarian axis is inhibited and release of hormones by a developed follicle is curtailed. The cessation of hormonal treatments results directly in low blood levels of oestrogen and progesterone and leads to a menstrual shedding of the endometrium (hormone withdrawal bleeding) whose development was attributed to exogenous oestrogen and progesterone. Thus, in this situation menstruation precedes the formation of a decidua in the fertile woman. However, this is a pharmacologically-contrived, rather than a naturally-induced, situation.

## The uterus role in birth

Foetal placentas are usually discarded at birth and we may have overlooked their orchestration of the uterine endometrium before parturition. As menstruation is coupled to shedding and regeneration of the endometrium, so is placental detachment from the uterus (47). An important feature of childbirth is the immediate repair of the endometrial surface attending placental detachment. The foetus not only arrests the maternal blood supply to the placenta but also stimulates tissue healing of the uterine surface. The same common processes appear early in menstruation leading to regeneration of the endometrium. The two processes are not, however, identical. In menstruation, blood flow in the spiral arteries in the endometrium is attenuated by their constriction under the influence of progesterone withdrawal. In a pregnant woman the walls of the spiral arterioles have been eroded and the chorionic villi are now bathed in maternal blood. In the last stage of pregnancy the large blood supply to the uterine placenta (48) via the uterine arteries (about a quarter of the cardiac output) has to be stopped for successful separation of the afterbirth to occur. Arresting this arterial blood flow at birth falls to contractions of the muscles in the adjacent myometrium induced by progesterone withdrawal which orchestrates onward local mechanisms that stop the uterine blood flow (3, 12, 13) (Figure 1). Should this process fail, massive haemorrhage (postpartum haemorrhage) would follow leading to what is still a major cause of maternal mortality across the world (49). There has been extreme natural selection for the maintenance of the genetic basis of this process across many generations. It is a vital component of motherhood.

The roles of progesterone, both in developing the endometrium prior to implantation and its withdrawal in initiating menstruation and parturition (3, 13), are central to this perspective article. Lowering the level of progesterone during the demise of the ovarian corpus luteum in the absence of pregnancy is physiologically comparable to progesterone withdrawal (however induced) by the foetal placenta during birth (50). Both induce the same contractile response by the blood vessels and myometrium, leading to either menstruation or successful detachment of the placenta from the uterus. From an evolutionary perspective, it is not possible to reconcile how the processes of foetal placental detachment that developed in all eutherian mammals to enable successful birth have been co-opted to initiate endometrial regeneration in humans. While Finn stated (21) that while menstruation *per se* may have no evolutionary future, the processes mediating placental detachment from the uterus represent the evolved and genetically-conserved condition in humans (51).

It may be appropriate to change the term “menstrual cycle” to “uterine cycle” whose initial phase is preparation for implantation, whose middle phase enables foetal development, and whose terminal phase is preparation for the onset of parturition. This would de-emphasize menstruation and emphasize the uterus' roles in embryonic attachment and birth. Then, in the absence of pregnancy, the significance of cyclical menstruation becomes apparent: it is the repeated anticipation of parturition and expression of the processes that determine foetal placental detachment. This change would add extra meaning to the term “decidua” (i.e., separation from a prepared surface) whose tissues, initially, nurture the embryo, but have their final role in placental separation.

## Discussion and considerations

Now that most human societies have achieved food security, cyclical menstruation will persist as a major factor affecting the long-term health and lives of women. Consequently, there is an associated greater morbidity of menstrual problems, such as debilitating heavy menstrual bleeding (often associated with uterine fibroids and adenomyosis) (3). However, menstruation indicates that women possess inherently the capacity for successful delivery of the placenta at birth. This capacity should be celebrated. The explanation, that menstruation and placental detachment share common, albeit not identical underlying processes that are vital for successful motherhood, should be the basis of education to reduce the stigma and prevailing social ignorance about menstruation. That menstruation is shown to have no essential biological purpose and is not needed for a healthy life may reassure women wishing to suppress problematic monthly periods. However, in diverse societies some view menstruation as an important natural process not to be interfered with, but whose debilitating signs are amenable to treatment.

The best evidence that menstruation is unnecessary for a healthy life is provided by women who suppress ovulation and their menstrual bleeding by using hormonal contraceptives (18, 52), and some professional athletes whose body fat level remains below their fertility threshold. However, their physiological capacity for menstruation remains. Similarly, should conception occur later, the same physiological processes are activated by the foetal placenta at the last stage of birth. A similar view was also expressed by Pavlicev and Norwitz (53) and Thomas (54). Progesterone withdrawal evolved as the initiator of parturition in all eutherian species, including humans (51). In human ancestors the suite of physiological processes involved with decidua shedding evolved in relation to parturition, with few appearances as menstruation. That ancestral condition has now been reversed with cyclical menstruation causing a woman to experience over 400 menstrual cycles in her lifetime (2). The explanation that cyclical menstruation developed as a consequence of enhanced human food security, and is relevant to the physiology of placental detachment at birth, remains highly plausible.

During the review process it was suggested that menstruation, while not essential for reproduction, could be indicative of a woman's systemic health. The concept of health includes interactions among the physical and mental parameters of a woman's life, all of which are not amenable to assessment via menstruation. Today there are many diagnostic tests and procedures that can accurately and promptly detect specific aspects of ill health. However, should a non-pregnant woman experience prolonged amenorrhea, medical intervention is necessary. Menstruation is indicative of serious systemic health issues. Globally, at least a quarter of women are afflicted by abnormal and/or heavy menstrual bleeding, together with the associated discomfort of abdominal cramps. This may also contribute to chronic undiagnosed anemia. It is critical that such women seek medical advice regarding treatment. These debilitating conditions may result in women not working, with an accompanying economic penalty to the woman, her family, and society. There is no simple solution for this form of ill health that may, in turn, affect adversely other aspects of a woman's health. This underscores the importance of both women and practitioners understanding the nature of menstruation and departures from its normal appearance. Women who choose hormonal treatment to prevent problematic or normal menstruation should be apprised of the risks from potential side effects relative to the benefits conferred and seek gynecological checks during this treatment.

Menstruation is emblematic of womanhood, and cyclical periods affirm that a woman is innately fertile and capable of successful childbirth. However, women may not appreciate being reminded of this each month. Globally, women must contend with the prevailing stigma, social ignorance, and the taboo attributed to menstruation in addition to the inconvenience it involves. No other aspect of femininity is subject to such mythology. For example, in some countries/societies menstruating women may be obliged to leave the household and not participate in domestic roles until the period has finished. Women and young girls living under financial constraints may not afford the regular costs of monthly hygiene products (period poverty) and choose not to attend school or college during their periods. This jeopardizes their education and potential to achieve, especially when compared to males. Policy changes on the part of governments (e.g., New Zealand and Scotland) can do much to resolve this specific problem, but such changes are so far in their infancy, both in affluent and lower-income countries. The indication of systemic illness that menstruation could indicate must be tempered by its detractions from women's health and wellness during their fertile life.

This examination indicates that there is much common ground between what is emerging as the disparate practices of Gynecology and Obstetrics. It is an opportunity to consider how understanding the mechanism of menstruation will further the understanding of parturition, also involving removal of progesterone (13) from the uterus. This interpretation of the non-essential role of menstruation in women's lives and reproduction should be the basis of further education and progressive regulation that promote women's health and welfare (4), especially aimed at reducing “period poverty” and menstrual absenteeism from schools and the workplace.

## Conclusions

Cyclical menstruation developed with assured food security and its related impacts on body fat, fertility thresholds, and ovulation. Proposed roles for menstruation were examined but shown to be biologically unnecessary for successful reproduction and a healthy life. Menstruation in a non-pregnant woman is an inevitable consequence of progesterone withdrawal. It is an alternate expression of the hormonally-controlled physiological processes that ensure placental detachment following birth. These vital processes determine survival of both mother and child and allow for further maternal reproduction. They are central to successful motherhood. Treating the adverse health effects of menstruation requires understanding the biological origin of this phenomenon and how it manifests itself in present day women. This perspective could offset the ignorance and myths surrounding menstruation, help develop a better scientific-medical understanding of menstruation's relation to women's health and be the basis of further education and progressive regulation that promotes women's health and welfare.

## Data Availability

The original contributions presented in the study are included in the article/Supplementary Material, further inquiries can be directed to the corresponding author.
